# A Review of Environmentally Friendly Approaches in Fire Extinguishing: From Chemical Sciences to Innovations in Electrical Engineering

**DOI:** 10.3390/polym14061224

**Published:** 2022-03-17

**Authors:** Hüsnügül Yılmaz-Atay, Jacek Lukasz Wilk-Jakubowski

**Affiliations:** 1 Department of Material Science and Engineering, İzmir Katip Çelebi University, İzmir 35620, Turkey; husnugul.yilmaz.atay@ikcu.edu.tr; 2 Department of Information Systems, Kielce University of Technology, 7 Tysiąclecia Państwa Polskiego Ave., 25-314 Kielce, Poland

**Keywords:** electrical engineering, firefighting, flame extinction, flame-extinguishing methods, acoustic oscillations, acoustic pressure, flame-retardant materials

## Abstract

After the invention of fire, the fight against fire probably started, and it has been going on for thousands of years. During this time, the aim has always been to extinguish the fire as soon as possible and to produce fire-resistant materials. Symmetry plays an important role in reducing problems, as it is a common feature of modern life. Multidimensional signal processing has many applications, an example of which is the use of appropriately timed acoustic waves to extinguish flames. This article provides a brief review of issues related to the use of acoustic waves for flame suppression based on studies in the literature. In addition, measurement results available in the literature obtained using a high-power acoustic extinguisher are discussed as a review of the scientific literature. Furthermore, we provide the latest information on the situation of flame retardants, including the latest innovations in basic sciences. In this sense, from intrinsically fire-resistant materials to flame-retardant additives and nanocomposites, new processes and applications are briefly mentioned.

## 1. Introduction

**Figure 1:**
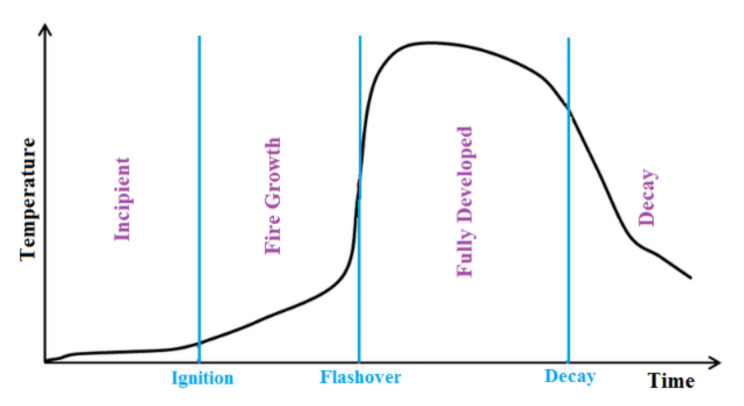
Traditional fire development.

Fire can be defined as a chemical combustion process involving the high temperature oxidation of a flammable material (or a fuel). As a result of this reaction, energy is released and heat is produced. Oxygen, temperature, fuel, and chemical reactions are required for the fire to start. These four elements are called a fire tetrahedron. For the fire to continue, the oxidizer and fuel supply must continue. Consequently, the heat given to the environment in the combustion reaction allows the fire to preserve its heat and ensures that the chain reactions required for the spread of the fire take place [1,2,3]. Figure 1 shows the traditional fire-development stages. Accordingly, no flame is visible in the first stage, and the smoldering phase occurs. In the second stage, ignition occurs. With well-ventilated flaming, fire growth occurs at this stage. New fuel or the right fuel and oxygen mixture is required for the fire to develop. At the flashover point, the fire spreads by reaching sufficient oxygen, fuel, and heat. After the fully developed phase is reached, the available fuel is exhausted and combustion ends. Those who try to extinguish the fire after the flash exceeds the level experience greater difficulty [4].

In the fire-extinguishing process, in principle, one of the components in the fire tetrahedron must be removed. For example, a natural gas fire occurring on a stovetop burner can be extinguished in various ways: Eliminating the fuel source by shutting off the gas supply may be the first option. Another option is to completely cover the flame to block any oxidizer that is present, such as oxygen in the air, during combustion. Another alternative is the application of water that quickly removes heat from the fire. Blowing hard on a flame can be considered a similar practice in principle, as it will shift the heat of the already burning gas away from the fuel source. Another option is to apply various chemicals that delay the chemical reaction in fire until they slow the rate of combustion [1,2,3,4].

## 2. Fire Extinguishing

Currently, flames are extinguished with the use of fire extinguishers that contain appropriately selected chemicals. The cost of their purchase is significant, and it has been noticed that they have a negative impact on the environment and human health. The best example seems to be the halon extinguishers, which are withdrawn from production because of their harmful nature. Halon extinguishers are now being replaced by so-called pure extinguishing agents, which are appropriately selected extinguishing gases. According to the NFPA 2001 (National Fire Protection Association) standard, these agents include selected halogenated hydrocarbons and inert gases [5,6]. Although chemical technologies are well developed and widely used, their use is fraught with inaccessibility in areas of difficult access. In addition, they are invasive and their chemical composition is mostly toxic. Another drawback of using traditional fire extinguishers is the short operating time and the fact that when the extinguishing agent is exhausted, refilling of the tank can only be performed by professional services (this operation is time-consuming and transporting the extinguishing agent to the place of the fire is quite problematic). For these reasons, it is not standard practice to fill tanks with the extinguishing agent after it is exhausted, at the site where the flames are extinguished. Any delay in extinguishing the flames is of great importance, as spreading flames pose a threat to human life and health, as well as financial losses. It seems safer for the environment (usually when extinguishing a flash fire) to use fire blankets. However, when the flames spread, this method is dangerous, especially for people who are close to the source of the flames, because of the risk of burns and uncontrolled air blast. For these reasons, the use of this method is severely limited [7]. 

Recently, the search for new, environmentally safe, and inexpensive methods of fighting fires has become a particularly important topic. Controlling and steering the flame has many different applications, despite the complicated nature of this process. In this area, there have been numerous studies carried out in recent years in Europe and around the world, which can be exemplified by the growing number of publications related to this topic. Some authors proposed the use of the acoustic field to reduce the emission of undesirable combustion products, such as soot, or presented the application of acoustic waves in industry [8,9]. The rapid development of technology in the 11th century also allowed the use of physical phenomena accompanying the propagation of acoustic waves for flame extinguishing. This approach could provide an alternative or complement to available flame control and suppression methods in the future and could revolutionize the currently available firefighting measures. Research in this area was carried out by the U.S. Defense Advanced Research Projects Agency (DARPA), starting in 2008 under the Instant Fire Suppression Program (IFS) [10]. The reason for initiating this program was the fire on the aircraft carrier USS “George Washington”, which within 12 h caused material losses estimated at $70 million. The escalation of this event led to strategic action. Taking into account the shortcomings of traditional means of fire protection, the need to look for new ways to combat uncontrolled phenomena, which are fires, was noticed. In addition, the solutions found in the following are relevant in this regard: (A) No. 4,735,282—“Device and circuit for the generation of vortex rings” (5 April 1988); (B) No. 5,899,685—“Remote lighted wick extinguisher” (4 March 1999); (C) No. 2010/0203460—“Process of extinction, expansion and controlling of fire flames thru acoustic” (12 August 2010) [11,12,13,14]. Furthermore, the “Mythbusters” in 2007 proved that flames can be extinguished using an appropriately amplified and modulated human voice (they achieved a sound pressure level that exceeds the pain level of the human ear) [15]. Acoustic waves were also used to extinguish flames a few years ago by two American students, Seth Robertson and Viet Tran. After a year of experimentation, they built a device that is capable of extinguishing flames [16].

**Figure 2:**
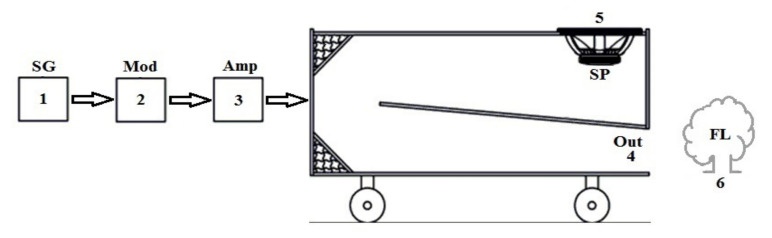
Components of an example acoustic extinguisher: (**1**) signal generator, (**2**) amplitude modulator, (**3**) amplifier, (**4**) device output, (**5**) acoustic wave source (speaker), (**6**) flame source.

Based on the literature review, the first attempts to extinguish flames using acoustic waves in Europe took place in the 1990s. This topic was again discussed in 2017 in Poland after obtaining a grant co-financed by the Ministry of Science and Higher Education from the “Innovation Incubator +” program (project no. 3 entitled “Non-invasive project of acoustic extinguisher using natural mechanisms of sound waves propagation to extinguish liquid fires in closed spaces”) [17,18]. Three patents and one small patent (utility model) can be identified as the results of this research [19,20,21,22]. In the conducted experiments, scientists showed that acoustic waves may be used to extinguish, inter alia, burning gases. In practice, the range and effectiveness of the acoustic flame-extinguishing technique depends on the acoustic flux density. An example of a high-power acoustic extinguisher is shown in Figure 2.

In 2020, the Polish and Bulgarian media published many articles discussing the possibility of acoustic flame extinguishing [23,24,25,26].

Since the technique of extinguishing flames using acoustic waves is a novel technique, few studies can be found in the literature that present the state-of-the-art in this field. In this article, due to the limitations relating to the areas indicated (Central and Eastern Europe), the following sections focus primarily on the work of researchers from this area. As a complement to the state-of-the-art, the works of other authors from outside the old continent (especially from America and Asia) who have taken up this topic are also discussed.

### 2.1. State of the Art in the Use of Acoustic Flame-Extinguishing Technology

There are known studies in Central and Eastern Europe concerning the possibility of using very low-power acoustic waves to extinguish flames a short distance from the device output, e.g., [27,28]. In the available literature, we can find several articles related to experimental research of flame behavior as a result of the acoustic field (articles [29,30,31,32,33] may serve as examples). There are few articles and scientific papers devoted to the potential use of high-power acoustic waves for flame extinguishing (this technology is relatively new and is still in the testing phase). This technique is particularly suitable for extinguishing firebreaks (in the first phase of the origin of the fire) [7]. In practice, the effectiveness of firefighting action using the acoustic method depends on the amplitude of air vibrations, which differs according to the sound source applied. The causal factor is the movement of particles forced by sound with the power necessary for this phenomenon to occur. Due to their physical properties, low-frequency sounds propagate uniformly in all directions and enter into hard-to-reach places, which is an unquestionable advantage. It seems desirable to explain this phenomenon. Propagated acoustic waves, through the transmission of medium disturbances (changes in air pressure), affect the flames with energy proportional to the value of the sound intensity, which defines the average value of the acoustic energy stream that flows through a unit of surface perpendicular to the direction of wave propagation [34]. For practical reasons, the notion of the sound intensity level expressed in dB may be used. Waves with low frequencies and high sound intensity are perceived as vibrations. The action of acoustic waves on flames results in the following: tearing them apart when the critical frequency is reached, dispersion (weakening), and extinguishment. This is possible when the temperature of the torn-off portion of the flame is lower than the ignition temperature of the flame portion. The mechanism of flame stream rupture due to acoustic wave interaction is discussed in detail in the paper [35].

**Figure 3:**
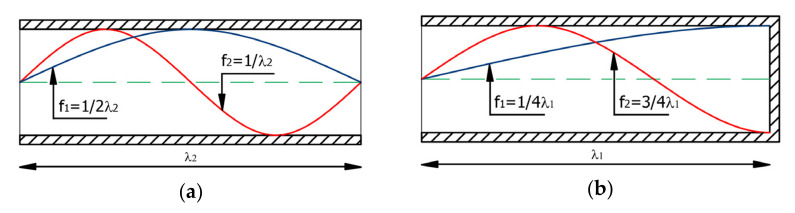
Standing wave distribution for the first and second resonant frequencies in an open tube (**a**) and a closed-end tube (**b**).

By analyzing the literature review, it was found that different types of flames have been extinguished in an open environment or inside a resonant tube. The simplest waveguide can be a closed and not very practical to use (due to its dimensions) tube with a circular cross-section. In addition to cylindrical tubes, other waveguides may also be useful, such as exponential or conical tubes. A characteristic of tube resonators is that their transverse dimensions are much smaller than the wavelengths at which they amplify. The acoustic wave is amplified as a result of the resonance phenomenon occurring in the resonator due to the reflection of the acoustic wave inside the waveguide and the overlapping of waves. A standing wave, whose position does not change in space, is created as a result of the overlapping of waves that move in the same direction but have opposite returns. Then, the amplitude of waves of a certain frequency is amplified. The distribution of standing waves (Figure 3) is given and discussed in [36].

The point where the vibration amplitude is equal to 0 (node) at the open end of the tube, which is the output of the device (at this point, the sound pressure reaches a minimum value), is marked in red (these considerations apply to Figure 3 on the left side). On the other hand, the point where the vibration amplitude is maximum (arrow) at the closed end of the tube is marked in blue (at this point, the sound pressure reaches a maximum value). As can be seen in Figure 3, the sound pressure decreases with the increase in the distance from the closed end of the tube (the minimum pressure level is noted at the open end of the tube). In Figure 3 (right side), the nodes at the open ends of the tube are marked in red. The end of the waveguide can be considered as a point source from which a wave of frequency equal to the frequency of the standing wave generated in the waveguide is propagated [8]. In addition, the use of a diaphragm allows one to increase the sound pressure value pointwise (the increase in sound pressure is noted when the diameter of the device output is reduced). Because the required waveguide length is twice as small in a closed-end tube than in an open tube, the closed-end tube is typically used for practical applications.

In the literature, experimental results may be found to illustrate the flame behavior due to the interaction of acoustic waves. Depending on the frequency, it can be observed that the acoustic field may be both linear and nonlinear. In practice, the sound pressure increases linearly only in a certain range, depending on the electrical power applied to the sound source, and the combustion may have a discontinuous pattern [34,35,36]. Some works have described unpredictable wrinkling [37,38] or bifurcation [39,40] of the flame.

Im, Law, and Axelbaum proved that flame extension due to turbulent vortices results in an aerodynamic distortion of the flame, which consequently leads to flame extinction [35]. The turbulence values of one of the ambient parameters interact with the propagation velocity of the flame front, affecting the change in its direction. Then, the flame extends, which results in increased heat emission until the flammability limit is exceeded [28,41,42].

Taking into account non-European studies, McKinney and Dunn-Rankin used methanol located in front of the output of the resonating tube as a fuel [43]. They recognized that the acoustic pressure required to note the extinguishing effect is dependent on the frequency and diameter of the methanol droplet. Therefore, they analyzed the extinguishing capability for different droplet diameters, frequencies, and sound pressures. The authors showed that increasing frequency results in better mixing between the fuel and the oxidizer, and consequently, a higher sound pressure is required to extinguish the flames [43]. In other words, when the flame is moved far enough away from the methanol droplets, the fuel supply resulting from the evaporation process is cut off. As the droplet size increases, the sound pressure needed to burn the flame increases. By comparing the operating frequency and flame power, it is expected that a thin (high) flame is easier to extinguish than a wider flame. From this, it can be concluded that flame inertia is also an important factor in determining the level of minimum sound pressure at which the extinguishing effect may be observed.

Beisner et al. used a lit Zippo lighter in microgravity as the flame source for their experiments [44]. They applied a one-ton acoustic wave for extinguishing. These authors concluded that it is easier to extinguish the flame using low-frequency acoustic waves, especially in microgravity, than in an ordinary gravitational field.

In turn, Bennewitz et al. [45] included extinction criteria for single drops of three different fuels. 

On the other hand, Friedman and Stoliarov, in their research, attempted to explain how acoustic perturbations affect flames with a laminar diffusion line [46]. Another research objective was to determine the necessary conditions to extinguish the flames. Similarly to the other authors, it was found that it is easier to extinguish the flame using low-frequency acoustic waves (as the frequency increases, an increase in the minimum sound pressure necessary to extinguish the flames is noted). In addition, it has been observed that as the acoustic pressure increases, the average mass loss rate of the fuels increases. In practice, the extinction process is associated with a temporary increase in the local flame deformation rate as the flame moves toward the droplet surface [46].

In the vast majority of studies, liquid fuels were used for the generation of flames. It is noticeable that, apart from the studies conducted in Poland, there is practically no research on the possibility of extinguishing flames using gaseous fuel, e.g., [17,47,48,49,50]. There is a lack of experimental studies that contain information on the influence of the distance of the device output from the flame source, produced with gaseous fuel, on the extinguishing process. It is worth noting that when gaseous fuel is used, unlike liquid fuel, the evaporation process does not occur. Research conducted in Poland has focused primarily on determining the minimum level of sound pressure and power delivered to the sound source at which the extinguishing effect is observed, depending on the frequency of the acoustic waves and the distance between the source of the flame and the device output. 

The possibilities of extinguishing a diffusion flame using the acoustic technique, taking into account the critical frequency value and the limit of acoustic power, are presented in [28]. The extinguishing phenomenon was visualized by means of a streak apparatus (a shadow method was applied using variations in the refractive index with a change in the density of the medium). In that research, it was shown that for a given value of the heat power of the flame, it is possible to extinguish it with acoustic waves of different frequencies (35 ÷ 155 Hz) for a power of less than 30 W. The highest extinguishing capacity was recorded for low frequencies. This paper also provides information on the flame extinction efficiency coefficient derived from the ratio of the limiting power and the thermal power of the flame with an appropriate multiplier (10^−4^ m^3^). As shown, this coefficient is functionally related to the critical frequency of the acoustic wave [28]. 

Niegodajew et al. [47] used acoustic waves in the frequency range (30–50 Hz) for extinction, for different burner powers and at different flame-source distances from the device output. The operating frequency range was chosen in a noncoincidental way. It was stated in [48] that for acoustic waves with frequencies below 100 Hz, the combustion rate decreases. Confirmation of this fact can also be found in some other publications, such as [43], where it was shown that the easiest way to extinguish the flame is to apply the lowest of the frequencies analyzed within the range of 75–135 Hz. On the other hand, it was suggested in [46] that a further reduction in frequency has a beneficial influence on the extinguishing effect. 

To the authors’ knowledge, there is a paucity of work in the literature that investigates the effect of various obstacles on flame extinguishment using acoustic waves. Niegodajew et al., in their paper [5], presented experiments showing how an acoustic screen affects the process of extinguishing flames with the use of acoustic waves (a single-obstacle model was applied). The experiment consisted of two phases, which were repeated three times to increase the accuracy of the measurements. In the first phase, the acoustic pressure required to extinguish the flames was determined. Two independent variables were analyzed, i.e., the fuel load and the distance between the acoustic screen and the device output. In the second phase, the acoustic field between the device output and the acoustic screen was studied. From the data presented, it can be concluded that the largest range of sound pressure variation was observed for a short distance from the acoustic screen (in this case 15 cm). Above this distance, a slight change in sound pressure values was observed (the use of a screen had little effect on the sound pressure level). This was evident regardless of the power delivered to the sound source. In the case of a close location of the screen and a probe, a systematic increase in the sound pressure was observed throughout the measurement range, regardless of the distance between the device output and the screen. The authors concluded that the direct environment affects the ability to extinguish flames using acoustic waves [5]. In a situation where there was an object directly behind the flame, the process of extinguishing the flames despite the increase in acoustic pressure was hindered. The closer the object was to the flame, the greater the sound pressure that was required to extinguish the flames; thus, it can be noted that increasing the sound pressure alone is not crucial to extinguishing the flames [5]. The mean flow effect is also a causal factor that impacts the flame-extinguishing process. To visualize the flame behavior under the influence of acoustic waves, the Schlieren method was applied. According to Chen and Zhang and Niegodajew et al. [30,47], the mean flow effect is independent of the excitation frequency and increases due to an increase in acoustic pressure. Based on the camera images, we infer that the flame itself follows the fuel flow until it is detached from the burner output. This means that the mean flow causes a deflection of the fuel jet and affects the separation of the flame from the burner output by moving it from its original position. When the critical sound pressure is reached, the flame is interrupted. On the basis of this, it may be concluded that flow and oscillatory disturbances affect flame extinguishing [47].

The authors of [27] provided information on extinguishing the combustion process with the use of acoustic waves, which have a nondestructive effect on protective objects. As highlighted by the authors, the ideal solution seems to be the usage of acoustic waves for firefighting, especially as elements of fixed firefighting systems. This long-term approach can be an alternative or complement to the classical methods of fire protection. As stated by the authors, in the case of generating acoustic waves with frequencies from 20 Hz to 5000 Hz, when the power applied to the loudspeaker was less than 30 W, the largest amplitude of change in sound pressure for the tested system was achieved for the frequency of 40 Hz [27]. For these experiments, depending on the frequency, the power difference at which the extinguishing effect was observed varied by 17 times at three-and-a-half times the acoustic pressure difference [27].

In the case of many experiments conducted in Poland using high-power acoustic extinguishers, the flame sources were candles placed in the axis of the waveguide behind the device output, as well as a professional gas-fueled mock-up that was used in practice to evaluate the effectiveness of firefighting actions carried out with traditional fire extinguishers (they were placed in both longitudinal and transverse positions in the axis of the waveguide). The operating frequency of the experiments oscillated around 17.25 Hz and was chosen in a noncoincidental way because the minimum acoustic impedance of 11.4 Ω was recorded for this frequency [36,49]. This was achieved by analyzing measurements of the experimentally derived impedance curve of the extinguisher, in the range from 10 Hz to 90 Hz. For the determined frequency, the vibrations of the speaker’s cone were smallest, which allowed the effective use of the power of the sound source. It was shown that it is possible to extinguish flames using multiple frequencies, both lower and higher than the operating frequency, while the distance of the extinguisher from the flame source is also important. At low frequencies, the more turbulent effects of acoustic waves on the flames were noted (the vibration amplitude increased significantly). However, frequency mismatch may be associated with substantial vibrations of the loudspeaker diaphragm (design limitations of the sound source). On the other hand, the emission of acoustic waves at a frequency higher than the operating frequency affected the increase in electrical power delivered to the loudspeaker to extinguish the flames (subacoustic power energy increases) [49]. From the point of view of an extinguishing action carried out with the use of an acoustic system, it is desirable to produce a sound pressure at a level sufficient to quickly extinguish the flames while maintaining a minimum sound pressure reserve (the term sound pressure reserve is understood as the excess sound pressure added to the minimum pressure at which flame extinguishment was observed). Therefore, the reserve is a correction for flame-extinguishing conditions that are different from laboratory conditions [49]. The author showed that variable-frequency acoustic waves can be used to extinguish flames, which may have a potential significance in suppressing flames originating from different materials.

It is worth emphasizing that fire locations usually have different concentrations of volatile substances such as CO_2_, smoke particles, O_2_, CO, NO_x_, etc. Since acoustic waves pass through solids, liquids and gases, according to the theory, acoustic extinguishers can be effectively used to extinguish fires of different classes [49]. This is because the acoustic field has a point effect on the flame source. In practice, it is possible to adjust the frequency and amplitude of vibration of acoustic waves to the flame source, which makes it possible to note that acoustic extinguishers are universal in contrast to classical fire-protection measures. It is also possible to use acoustic waveforms with different modulation and varying frequency; for example, when the composition of the substance is not exactly known [49]. According to the combustion triangle, which denotes the condition of fire formation, the composition of a fire includes the following: oxidant, heat and combustible material. From the point of view of flame extinguishing, it is important to generate waves with a frequency chosen to locally repel oxygen molecules from the air. Besides the frequency, the acoustic power is an important parameter, which influences the effectiveness of the acoustic-wave extinguishing process. It has been proven that the effectiveness of the extinguishing process depends on the amplitude of vibration of acoustic waves. The sound wave increases the speed of air movement at the periphery of the flames, thus reducing the area in which the combustion process occurs. This can be seen perfectly in the video presented by DARPA [10,36,50]. Thus, we have two dynamics (the acoustic field increases the air velocity, which disturbs the flame boundary layer; in turn, the disturbance of the flame surface leads to more vaporization of the fuel, which affects the expansion of the flame, thus lowering its overall temperature). The acoustic method thus appears promising for flame extinguishing when access to conventional extinguishing agents is limited or when the composition of the substance is unknown. It is also worth noting that while harmful gases are emitted into the air during a fire when effective-though-harmful fire-protection agents (e.g., halon fire extinguishers) are used, acoustic waves are not a chemical product and do not pollute the air, which is a great advantage [7,49].

### 2.2. Possibilities of Using Deep Neural Networks Together with Acoustic Technology to Fight Fire

In the literature on the subject, there are works in which Deep Neural Networks (DNN) are used to determine whether a fire has occurred and, if so, the extinguishing system can be activated automatically. This is feasible if the acoustic extinguisher is equipped with an intelligent module. Communication is not a problem, especially when using wireless data transmission techniques (it becomes possible to remotely activate the acoustic system depending on the detection of flames by sensors and Deep Neural Networks without human intervention, which is important to protect against the effects of low-frequency waves on human health) [18,51]. This may find applications in robotics and emergency management [52,53]. Works carried out in Central and Eastern Europe, supported by institutions dealing, inter alia, with fire protection, resulted in the development of an acoustic system concept that is capable of extinguishing flames with the use of sound waves. Such a non-invasive acoustic extinguisher equipped with a flame-detection module with Deep Neural Networks may constitute an autonomous device capable of activating when flames occur. Research in this area is especially carried out as part of the cooperation between Polish and Bulgarian scientists.

Stawczyk et al. demonstrated the influence of air turbulence resulting from varying sound pressure on flame continuity for different distances from the flame source and multiple frequencies [36]. In practice, the effectiveness of the extinguishing process depends on the amplitude of the air vibration. Acoustic power and acoustic wave frequencies are important factors [7]. The acoustic extinguisher presented by the authors generates a directional acoustic flux to effectively extinguish flames.

For the frequency of 14 Hz, very good extinguishing efficiency was found with the lowest electrical power delivered to the extinguisher. The use of low frequencies forces a much longer waveguide. This is due to the fact that the lower the frequency, the longer the wavelength. As the frequency increases, the power required to extinguish the flames increases (the extinguishing range is affected by the concentration of the acoustic beam) [17]. On the other hand, the use of higher frequency acoustic waves allows for a reduction in the size of the acoustic extinguisher. The application range is determined by the frequency of acoustic waves, which is very important, especially in the case of acoustic waves with high acoustic pressure below the range of human hearing (infrasound). Due to their harmfulness, no human presence during extinguishing should take place. To perform quantitative analyzes, the classical regression function model can be used in the linear range [54].

The subject of recent research, however, is the possibility of connecting an acoustic fire extinguisher to smart sensors and using Deep Neural Networks to determine whether the flames have been detected, that is, whether a fire has occurred. Ivanov et al. presented the beginnings of the use of acoustic techniques to extinguish flames [50]. The authors pointed out that current methods of extinguishing flames involve the use of solid, liquid, or gaseous extinguishing agents. The acoustic method works by increasing fuel evaporation and dispersing the flames over a large area and results in the reduction in flame temperature and the area over which the flames occur. The velocity of air movement at the edge of the flames increases, while the density of the acoustic jet affects the operating range of the acoustic extinguisher. This paper demonstrated the feasibility of using Deep Neural Networks for flame detection based on learning from images or video streams [55,56,57,58,59,60]. Such a system, consisting of a computer module, a USB camera, and a Movidius stick vision processing unit, may be connected to an acoustic fire extinguisher and can allow it to be activated when the flames are detected. The hardware is based on a Raspberry Pi board, which allows support for multiple operating systems. The controller is capable of generating complex video signals, including 576 i and 480 i for PAL, NTSC. A USB camera is connected to the Raspberry Pi (the resolution is 1280 × 720 pixels). Two relay modules (24 V control signals) can be applied to control the fire extinguisher depending on the detection of flames in the captured video stream. Many technologies and libraries are implemented in the control software, the most important of which are: OpenCV (computer vision and machine learning library), Matplotlib (separating library), TensorFlow (for machine learning), and NumPy (for handling multidimensional arrays). The neural network is based on the MobileNet architecture, designed for mobile and embedded applications. The described technology was also presented in [17]. Many other libraries, such as Imutil [7], may be useful as an auxiliary tool for the implementation of neural networks for flame suppression. These networks can be implemented in various processing systems (CPU, VPU, and GPU).

**Figure 4:**
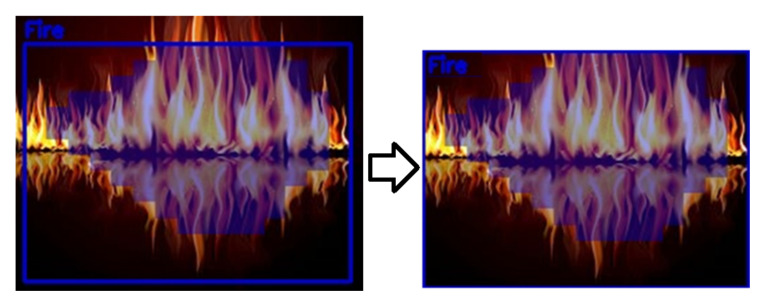
Dominant fire source image and flame detection.

Publication [7] describes a development kit consisting of a System on Module (SoM) and a reference backplane (the purpose was to develop computationally powerful embedded systems). The board has GPIO pins to control the acoustic flame-extinguishing system. An example of flame detection is illustrated in Figure 4.

The neural network presented is reliable in flame detection, so it can be integrated into modern fire-protection systems (without the use of additional sensors) [7]. The acoustic system can also be connected to smoke and temperature sensors, so human presence is not required during extinguishing (the extinguishing system is able to be activated automatically).

The authors of [17] presented a method for extinguishing flames with a high-power acoustic extinguisher using modulated and unmodulated acoustic waves (the sound does not have to be loud; the important thing is that it is appropriately modulated). The extinguisher is equipped with a flame-detection module using artificial neural networks. These networks were learned using still images and video streams. Such an intelligent module applied in an acoustic fire extinguisher can support fire protection implemented using acoustic flame-suppression technology [49]. This is a new method, as the acoustic extinguisher equipped with a fire detection module using Deep Neural Networks is an innovative approach in fire protection as a result of Polish–Bulgarian cooperation (between the Kielce University of Technology and the Technical University of Gabrovo). The effect of this research is a fire-extinguishing system with high and very high acoustic power, and the artificial intelligence platform. Sinusoidal waves modulated with a triangular waveform (AM modulation was applied in this case), and unmodulated triangular waves were used to extinguish the flames [17]. For the sinusoidal waveform modulated by a rectangular waveform (AM modulation, MFreq = 0.125 Hz = const.), the results for the electrical power delivered to the sound source (required to extinguish the level of flames) and the sound pressure level as a function of distance from the output of the extinguisher were provided in [7]. Ivanov et al. showed that AM-modulated waveforms are effective for flame extinguishing [50]. Sinusoidal waves modulated by triangular waveforms may also be applied for this purpose. The results presented in this paper showed the sound pressure level at which the flames were effectively extinguished as the function of a distance from the extinguisher output, ranging from 50 cm to 130 cm. An inversely proportional relationship was experimentally demonstrated between a decrease in sound pressure level and an increase in the distance from the extinguisher output. This means that the greater the distance from the flame source, the higher the electrical power that has to be delivered to the sound source to extinguish the flames.

The authors of [18] provided results for a system that can operate as a standalone platform and work with other hardware modules, such as Arduino. This platform is equipped with a 16-bit processor for Deep Neural Network processing as well as two 64-bit RISC-V processors. A Kendryte K210 processor was applied for flame detection. If they are identified, the control of the acoustic extinguisher is applied discretely. The advantage of the system is its high processing speed (the computation time is about 10 ms) [18]. This paper reports results on the flame-extinguishing capabilities of a high-power acoustic extinguisher for a sine wave and a sine wave modulated by a rectangular waveform for several analyzed frequencies (15, 17, and 20 Hz). These networks are useful for flame detection in various equipment, buildings, or transport (land, water, and air), especially where, for example, ambient temperature and the presence of dust prevent or significantly influence the efficiency of using other types of flame-detection techniques [18]. In this respect, foundries, sand dryers, and heat treatment plants, inter alia, can be pointed out. Moreover, acoustic flame-extinguishing technology may find application for extinguishing flames of substances whose properties make it impossible to extinguish them by conventional means or wherever access to conventional fire-protection agents is severely limited (e.g., in aircraft, trains, ships, etc.). In addition to the availability of many technologies, the low cost of the components used and the high efficiency of flame detection should be mentioned as the advantages of flame detection using neural networks instead of traditional sensors [7].

The authors showed that it is possible to construct an acoustic system consisting of multiple extinguishers and take advantage of the natural mechanisms that accompany the propagation of sound waves (constructive interference) [17]. Furthermore, interference screens, acoustic baffles, and panels, as well as many materials that absorb the harmful part of the acoustic wave energy in order to obtain additional gain, may be applied. This is important because low-frequency acoustic waves are poorly attenuated by the medium in which they propagate. Remote surveillance systems can be implemented using satellite communications. For this purpose, it is important to know the propagation phenomena that affect the attenuation of radio waves in the atmosphere of the Earth [61,62,63]. Other technologies described in articles by Croatian researchers [64,65] can also be useful for the transmission of data, from locations where a fire has occurred.

## 3. Fire-Resistive Materials

Looking at previous civilizations, even if they did not understand the physics and chemistry that took place, they seem to find ways to create fire retardants. It is seen that Egyptians used Alum to give wood non-flammability in 450 BC. The Romans used alum and vinegar for the same material in 200 BC. In the 1600s, fire-resistant canvas was used that produced a combination of clay and plaster. Clay and gypsum were used for theatre curtains in 1638 [66,67,68]. It is seen that a mixture of alum, ferric sulphate, and borax was used in wood and textiles in England in 1735. During these years, the first patent was received in England for flame retardancy of textiles using alum, borax, and vitriol. In the study by Gay-Lussac in 1821, a mixture of (NH_4_) PO_4_, NH_4_Cl, and borax was used to be effective on linen and hemp. This scientist used his chemistry knowledge on flame-retardant materials and took a serious step forward. He showed that when the mass and volume of a gas are kept constant, the gas pressure will increase as a result of the increase in temperature. This law is known as the Gay-Lussac Law. Correspondingly, the real evolution in fire-resistant research appears to have taken place in the late 1900s, with different scientists turning their research attention to this field. In the following sections, studies on fire-resistant materials will be mentioned [66,67,68].

### 3.1. Intrinsically Fire-Resistant Materials

Fire-resistant materials can be produced with various additives, while some materials are inherently fire resistant. Materials with very high melting temperatures can be examples of materials that are inherently fire resistant. However, the materials we use frequently in all areas of life are generally easily flammable materials, such as polymers. However, some polymers are classified as intrinsically fireproof materials due to their bonding structures. The high-temperature properties of polymers can be changed by increasing the interactions between polymer chains or by using chain-hardening methods. In this way, the degradation of polymers is possible at higher temperatures [69]. Chain interactions can be achieved in a variety of ways: increasing crystallinity, adding polar groups, hydrogen bonding, etc. For chain-hardening processes, aromatic or heterocyclic structures must be used in the polymer backbone. These structures are materials such as poly (propylene), aromatic polyamides, and polyesters. High thermal stability and less flammable volatile material production during combustion are sought in fire-resistant polymers. Consequently, polymers containing aromatic groups in their structures produce less flammable gaseous products during combustion.

In the work of Tong et al. [70], an intrinsically flame-retardant Nano-fibrillated cellulose was successfully prepared from kraft and unbleached bamboo pulps, respectively, through a three-step strategy involving quaternized pretreatment, mechanical disintegration, and subsequent borylation. The flame-retardant properties of the borylated Nano-fibrillated cellulose samples were comprehensively investigated in terms of microscale combustion calorimetry, limiting oxygen index, vertical burning, and cone calorimetry tests. A synergistic effect was observed between borylated and lignin species in promoting the flame resistance of Nano-fibrillated cellulose matrix. When used as flame-retardant coatings on filter paper and PET film, both borylated Nano-fibrillated cellulose films displayed excellent flame-retardancy and smoke-suppression effects. 

In another study, Nanosized cellulose nanofibrils (CNF) prepared from phosphorylated pulp fibers (P-CNF) were combined with CNF prepared from aminated fibers (cationic CNF) through a layer-by-layer (LbL) assembly to prepare a freestanding, transparent all-cellulose film [71]. It was shown that the thermal stability and flame-retardant properties of all CNF films are significantly improved when phosphorylated CNF is combined with cationic fibrils in an LbL-assembled structure. Although such studies are difficult and costly to synthesize, they are still ongoing. Various polyimides, polybenzoxazoles, polybenzimidazoles, and polybenzthiazoles appear as examples of polymers made with aromatic heterocycles [72,73].

### 3.2. Flame-Retardant Additives and Fillers

The easiest way to make a flammable material less flammable is by adding flame-retardant additives. Additives can be divided into two types, namely reactive and additive flame retardants. Reactive flame retardants, which are compounds containing heteroatoms, can be chemically built into the polymer molecule. Additional flame retardants can be mixed physically with existing polymers. In the mixture obtained in this way, there is no chemical reaction of flammable materials and flame retardants [66].

Considering the working mechanisms of flame-retardant additives, it can be seen that they work in the dense phase or the gas phase. It appears that in both phases, flame-retardant materials can move chemically and or physically. After pyrolysis, the heat in the environment produces gases. As soon as the required ratio between these gases and oxygen is reached, the material starts to ignite and burn [74]. In this case, there are three processes that can be seen in the dense phase. The first is the breakdown of the material, in which case the effect of the flame is reduced. The second is the carbon layer (char) formed on the surface of the material. The last is heat absorption through materials.

**Figure 5:**
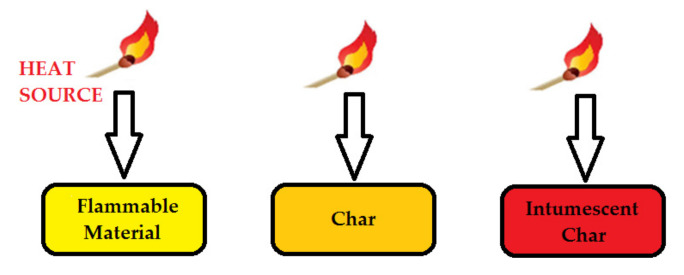
Char and intumescence formation.

Intumescence, a special case of condensed phase activity, occurs in the gas phase without the apparent involvement of radical trapping mechanisms. This can be defined as the increase in volume in the continuation of the coal formed. This layer plays an important role in dissipating heat and appears as an obstacle for oxygen to reach the combustible surface (Figure 5) [74]. Looking at the burning time in the gas phase, it can be seen that it is shortened by chemically interfering reactive species. This occurs when flame retardants interfere with free radicals formed during the combustion process. Consequently, reactions occurring in the gas phase (exothermic processes) slow down or even stop. As a result, the system cools down and limits the supply of flammable gases [74].

Hydrogen halides (HX, where X = Br or Cl) are formed by the reaction of halogenated organic compounds (R–X) with a polymer (P–H). They can react with the excited-state HO and H radicals. In this way, less reactive halogen free radicals, denoted by X, are produced. This leads to an overall decrease in the kinetics of the combustion. The reaction chain can be seen in the reactions below (Equations (1)–(3)) [74,75]:

R–X + P–H → HX + RP(1)

HX + H → H_2_ + X (2)

HX + OH → H_2_O + X (3)

When the combustion cycle is evaluated, substances such as H_2_O, H_2_, and HX are less reactive species. In the presence of flame repellents containing halogens in the environment, active radical species such as OH, O, and H can be quenched in the gas phase, resulting in the formation of less reactive species [13]. In the working mechanism of these additives, they first prevent free radical reactions in the dense phase. In addition, they act as heat sinks because of their heat capacity. They prevent combustion by cutting the contact of the flammable material with the heat source and oxygen by forming a fireproof protective coating. However, it is not possible to attribute a single mechanism to a single flame retardant. In general, fire retardants operate with different mechanisms depending on the nature of the inflammable substance [66].

#### 3.2.1. Halogen Additives

**Figure 6:**
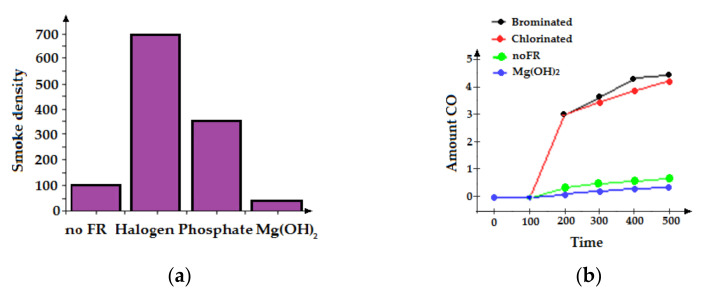
(**a**) Smoke density and (**b**) CO amounts of PP compounds with different flame retardants.

As mentioned briefly above, the working mechanism of halogen-containing fire-resistive materials occurs in the gas phase. The reaction produces toxic substances such as black smoke and CO, as well as incomplete combustion. Carbon monoxide is a very toxic gas that occurs in fires. It prevents oxygen transport to the blood and thus disrupts the respiratory process. Because of these toxic substances, flame retardants containing halogens (especially those containing bromine) are believed to increase human deaths from smoke inhalation in fires in North America. Smoke formation and the amount of CO emitted by polypropylene compounds containing different flame retardants can be seen in Figure 6 [75,76].

On the other hand, in the event of a fire, halogens are known to promote not only the formation of CO, but also the formation of other highly toxic substances such as dioxins and furans. In fact, these compounds bind to soot particles and do not directly kill people. However, these particles scattered throughout the building during the fire would be very difficult to clean. In fact, sometimes the only solution to cleanse the building of these particles is to rebuild [75,76].

#### 3.2.2. Inorganic Minerals

Halogen-free flame-retardant materials are known to be much safer than halogen-containing ones. Among these, those containing inorganic minerals are the most commonly used materials. Such materials decompose endothermically during fires, and water is released in this reaction. The fact that decomposition is endothermic allows heat around the flame to be removed. Thus, as a result of the cooling of the flame, pyrolysis decreases in the dense phase. The release of water as a reaction product causes the amount of oxygen in the environment to dilute, and this causes the fuel and oxygen ratio to move away from the critical level. If the ignition is blocked in this environment, it is especially necessary for some fire tests, such as those of electrical and electronic cable applications, to be conducted. Inorganic materials can be used in this sense. On the other hand, the ceramic layer formed following the decomposition reaction acts as a protective layer due to the insulation between the combustible surface and heat and oxygen, and its smoke-suppression feature [74,75,76,77,78].

Aluminum trihydrate (Al(OH)_3_) is one of the leading inorganic materials used as flame retardants throughout the world. This material, which entered the market in the 1960s, represents 43% of all flame-retardant chemicals. Although ATH is quite stable at room temperature, the decomposition reaction starts when it exceeds 205 °C. This temperature is a temperature that can be easily reached when a fire occurs. The decomposition reaction is endothermic and the reaction temperature is −298 kJ mol^−1^ [74,75,76,77,78]. In this thermal decomposition event, there is an endothermic water loss of approximately 35% caused by the mass of ATH. Studies have shown that the reaction is between 1170 and 1300 Jg^−1^. The ATH flame can be used as a retardant in materials handled below its decomposition temperature. This limits the application area of this material [68,78]. Taking into account the usage rate in composites, it is seen that this is 60% to obtain a good level of flame retardancy. This level worsens the mechanical properties of composites as a result of its high mineral content. This limits the use of similar inorganic minerals used in composites with such high loading [79,80].

Another inorganic flame-retardant mineral is magnesium hydroxide (Mg(OH)_2_). The flame-retardancy mechanism is similar to that of ATH. This mechanism involves releasing crystallization water during fire and thus restricting oxygen intake, which prevents the resulting oxygen deprivation from continuing combustion [81,82,83,84,85]. Magnesium hydroxide can be used as an additive material in the range of 40–65% by weight. Its decomposition temperature is 320 °C, and due to this feature, it is possible to use it in production processes with processing temperatures above 200 °C (for example, in the production of thermoset resins). This situation emerges as an advantage of magnesium hydroxide over ATH [75,76,77,78]. Its heat capacity is 77 kJ mol^−1^, which is lower than that of ATH, but it can absorb heat. When viewing the flame, it is seen that the gaseous water phase surrounds the flame. In this way, the flammable gases are diluted by excluding the oxygen. When it comes into contact with the flame, a thermal insulation material is formed on the surface of the flammable material. Therefore, this insulation material prevents the flow of flammable decomposition products into the gas phase where combustion occurs. This mechanism is similar to the function of coal, which occurs via the degradation of phosphorus-containing flame retardants. Products with these reactions are not toxic. Mineral phases of these products, e.g., MgO, are an alkaline product. In this way, the acidic and corrosive gases that may arise are prevented [86].

Huntite and hydromagnesite minerals entered the flame-retardant market in the late 1980s. This mineral, which degrades endothermically, has similarities to aluminum trihydrate and magnesium hydroxide in terms of its flame-retardant properties. With its endothermic degradation starting at 250 °C, it releases MgO and CaO as a solid, with the release of water vapor and carbon dioxide. PVC, cable, insulation, and construction products are the main areas of use. Huntite and hydromagnesite exist in nature in the form of a physical mixture. For this reason, this mixture is used directly as a flame retardant, without any separation process. The mixing ratio of the deposit normally ranges from 40% to 30% huntite and 60% to 70% hydromagnesite. Calcite and dolomite are generally seen as impurities. Taking into account their densities, huntite and hydromagnesite minerals have 2.70 g/cm^3^ and 2.24 g/cm^3^, respectively [75]. Huntite and hydromagnesite have many advantages in their use as flame retardants. For example, they create low smoke. They do not contain halogen, are non-toxic, and are not harmful to the environment. They are not corrosive to processing equipment. In addition, their cost–performance relationships are quite good [74,75,76]. In Table 1, the decomposition reactions of ATH, magnesium hydroxide, huntite, and hydromagnesite, which are mostly used in the sector, are given [77].

Phosphorus-containing flame-retardant materials are used for materials with high oxygen content, such as cellulose and some oxygen-containing plastics. When viewed as a mechanism, it is seen that in the condensation phase of the combustible material, the flame-retardant containing phosphorus is thermally transformed into phosphoric acid. Phosphoric acid draws water from the burning material and, accordingly, causes this material to char. The coal-like structure that is formed isolates the burning material from flame and heat, and stops volatile and flammable gases from escaping from the heap. Phosphoric acid is formed as a result of the combustion reaction, which increases the abrasiveness of the smoke [87,88,89].

Researchers have examined flame-retardancy properties in composites using additives such as borax and boric acid. Minerals such as tincalconite, colemanite, and ulexite are seen to be used as boron minerals [90]. Terzi [91] produced wood composites using colemanite and common boron-based flame retardants. For this, they used a mixture of zinc borate, boric acid, and borax. The study showed that boron-based compounds have a better fire-retardant property than colemanite [91]. In other studies, in which injection-molded wood flour or polypropylene composites were combined with boron compounds with different contents, borax/boric acid and zinc borate and phosphate compounds, and mono and diammonium phosphates, were used [92,93].

Boehmite, AlO(OH), which is a derivative of aluminum hydroxide, is seen to have a structure where two thirds of the water is removed when looking at its structure. Although it is seen in flame-retardant inorganic materials, it does not have a high performance. The reason for this is that the amount of water in its structure is low. However, it is on the agenda to use it with different types of flame retardants and fillers. In this way, better yields are expected [66]. Sun et al. synthesized Nano-boehmite added to bisphenol A epoxy resin to increase the flame retardancy of the resin in their study. In this way, they showed that boehmite can improve flame retardancy and Tg epoxy resin [94]. Huang et al. tried to increase the flame retardancy of Li-ion batteries by embedding the boehmite in the cathode. In their work, they observed that encapsulation with poly (urea-formaldehyde) remained a good flame retardant for boehmite. Encapsulated boehmite showed more than 40% fire retardancy without electrochemical sacrifice [95].

The material with the lowest cost compared to other materials is calcium sulfate dehydrate (Gypsum). Its flame-retardant performance is not very good. It starts to decompose below 100 °C, which is a very low value. However, it is known to be used as a flame retardant in unsaturated polyester resins [66]. Lee et al. investigated the burning properties of wood-based panels and gypsum chipboard using a cone calorimeter. In this context, they were measured in terms of their ignition time under a fire condition, heat-release rate, smoke-generation rate, and CO efficiency. According to their results, they found that the heat-emission rate, smoke-generation rate, and CO of gypsum chipboard panels were significantly lower than those of the wood-based panels [96].

Some of the above-mentioned inorganic minerals were used by us as flame-retardant candidates, and flame-retardant composites were obtained by adding them to the polymer matrix. In addition, in our studies, the possible synergistic effects of these minerals were investigated by mixing them in various combinations. One of the studies carried out in this regard is given below. In this study, which focused on a coating application, the plastic substrate was covered with a dye containing inorganic minerals. Huntite and hydromagnesite (HH), antimony oxide, and boric acid were utilized as flame-retardant minerals. Table 2 shows the minerals used, the amounts of additives, and the sample codes.

**Figure 7:**
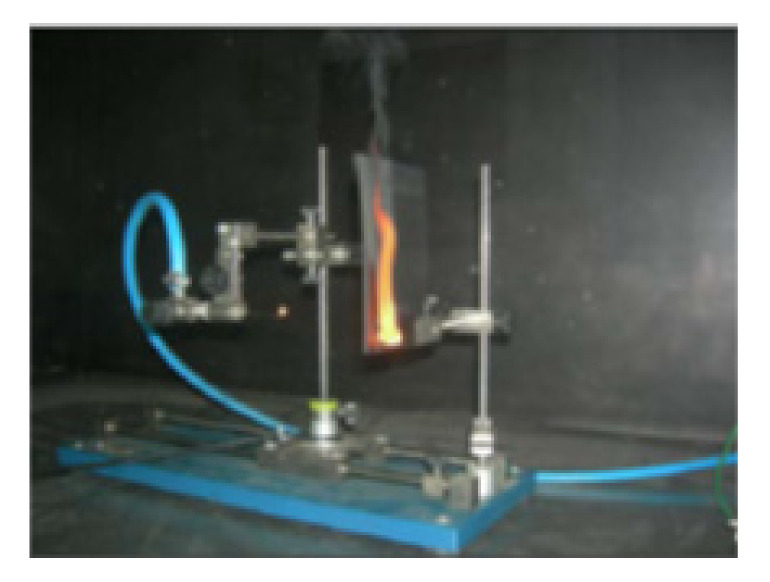
The UL-94 needle flame test set up.

Flame-retardance tests of the obtained samples were carried out according to the UL94 standard. According to the UL94 test, the material is directed towards the flame at a specific angle and distance. The extinguishing time of the flame and the dripping ability are measured. Figure 7 shows the set-up of the UL-94 needle flame test. The samples were subjected to flame for 3 min after the calibration process was completed (flame height: 12 mm; it took 23.5 s ± 1 s to reach from T1 (100 °C) to T2 (700 °C)). The gas used was propane with 99.5% purity.

**Figure 8:**
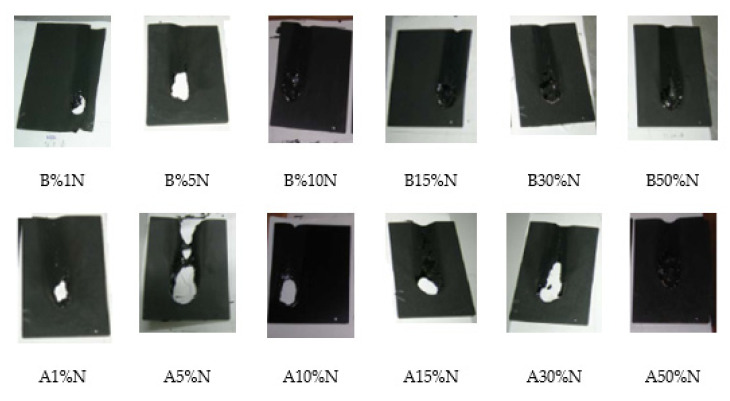
Candle flame test results of the composite coatings.

The test results are demonstrated in Figure 8. The figure depicts antimony oxide-huntite hydromagnesite and boric acid-huntite hydromagnesite-reinforced composites. More specifically, as the amount of boric acid in the structure increases, the performance of its flame retardancy increases. Furthermore, Figure 8 also shows the flame-retardant properties of samples coated with antimony oxide-huntite hydromagnesite-reinforced polymeric composite.

It can be seen that the flame-retardancy performance increases with increasing amounts of antimony oxide in the composites. Generally speaking, when the amounts of boric acid and antimony oxide are increased in huntite hydromagnesite-reinforced polymeric composite coatings, their flame-retardant properties are strongly improved. What is expected from the use of flame retardant in paints as a binary system is to provide outstanding properties. Synergetic beneficial effects are obtained with binary flame-retardant additives in the composite coatings [97].

### 3.3. Nanocomposites

Nanocomposites differ from conventional composite materials due to the exceptionally high surface-to-volume ratio of the reinforcing phase and/or its exceptionally high aspect ratio. They are emerging as a new class of flame retardants. Looking at the additives used in flame-retardant nanocomposites, it can be said that they include a wide range of materials ranging from inorganic materials to graphene, carbon nanotubes, boron nitride nanosheets, and MXenes.

Weil et al. produced 5% clay containing polyamide-6 clay nanocomposite. In addition to flame retardancy, an increase in the strength value of the composite was observed in this material [98]. In another study, Blumstein [99] synthesized poly (methyl methacrylate) in the presence of clay and discovered that clay has a stenciling effect on polymer formation. When looking at flame-retardant nanocomposites, it is seen that most of them are generally obtained with clays. Most studies are conducted with montmorillonite clay, which is an alumina-silicate material [100,101,102,103]. Carosio et al. [100] produced cellulose nanofiber (CNF)/clay nanocomposites with a unique brick–mortar structure that were prepared by simple filtering in order to eliminate problems caused by toxic materials encountered in some flame-retardant additives. As a result of this work, they found that these nanocomposites have superior fire-protection properties compared to other clay composites and fiber composites. However, we also see that graphite and carbon nanotubes are used. The difficulty with single-walled carbon nanotubes, which are more expensive and are newly studied, is the need to organically modify the nanotubes. The reason for this is that nanotubes are organophilic. This is not required for multi-walled nanotubes [104].

Graphene, a carbon nanomaterial with unique properties, appears in new-generation flame-retardant materials. Graphene sheets have an effective ability to form a protective char layer as flame retardants. It was observed that these boards have a superior flame-retardant effect as compared to clay and CNTs [105]. Wang et al. [106] worked on the functionalization of graphene with grafted polyphosphamide for flame-retardant epoxy composites. In this study, a polyphosphamide (PPA) was synthesized and covalently grafted onto the surface of graphene nanosheets (GNSs) to obtain a novel flame retardant, PPA-g-GNS, and subsequently PPA-g-GNS was incorporated into epoxy resins (EPs). The evaluation of the thermal properties demonstrated that the addition of PPA or PPA-g-GNS to epoxy had a thermal destabilization effect below 400 °C, but led to a higher char yield at higher temperatures. The PPA-g-GNS/EP composite exhibited superior fire-resistant performance.

Xing et al. used functionalized carbon nanotubes with phosphorus- and nitrogen-containing agents to improve the flame resistivity property of polystyrene nano-composites. In their study, aminated multiwalled carbon nanotubes (A-MWCNT) were reacted with diphenylphosphinic chloride (DPP-Cl) to prepare the functionalized MWCNT (DPPA-MWCNT). The incorporation of DPPA-MWCNT to PS significantly reduced the peak heat-release rate, smoke-production rate, and carbon monoxide and carbon dioxide release in cone calorimeter tests. Looking at the flame-retardant mechanism, it can be said that the char layer works as an insulating barrier to reduce the exposure of the polymer matrix to an external heat source and to delay the feeding of flammable gases to the flame [107].

Wang et al. [108] used multifunctional boron nitride nanosheets to reduce the toxic volatile (CO and HCN) generation and fire hazard of thermoplastic polyurethane. They prepared the multifunctional CPBN via the wrapping of the phytic-acid-doped polypyrrole shell, followed by the adsorption of copper ions. They showed decreased peak heat-release rate, peak smoke-production rate, and total smoke-production values. The suppression of CO and HCN release was also achieved.

Yu et al. used exfoliated MXene ultra-thin nanosheets for the fire and smoke suppression of thermoplastic polyurethane elastomer. They proposed a simple method for the preparation and utilization of cetyltrimethyl ammonium bromide (CTAB) and tetrabutyl phosphine chloride (TBPC)-modified Ti_3_C_2_ (MXene) ultra-thin nanosheets. Significant reductions in the peak heat-release rate (51.2% and 52.2%), peak smoke-production rate (57.1% and 57.4%), peak CO production (39.4% and 41.6%), and peak CO_2_ production (49.7% and 51.7%) were recorded by the mere introduction of 2 wt.% CTAB-Ti_3_C_2_ and TBPC-Ti_3_C_2_ to TPU during the cone calorimeter tests. The properties obtained in this study, resulting from the significant reduction in fire, smoke, and toxicity hazards, were due to the excellent dispersion, catalytic, and barrier effects of Ti_3_C_2_ ultrathin nanolayers in TPU [109].

Zhu et al. [110] utilized BODIPY coated on MXene nanosheets to improve the fire safety properties of ABS resin. In this study, boron dipyrromethene (BODIPY)-modified MXene (Ti_3_C_2_Tx) nanosheets were prepared and utilized as a flame retardant for ABS. The limiting oxygen index (LOI) value was increased from 19.5% for neat ABS to 21.5% and 23.5% for ABS/BODIPY-MXene0.5, and ABS/BODIPY-MXene2.0 benefitted from the rapid carbonization. The improvement in fire safety was achieved due to the excellent barrier, free radical capture, and catalytic carbonization effect of the BODIPY-MXene nanolayers in the ABS matrix.

### 3.4. Flame-Retardant Coatings

Coatings are very effective in imparting superior properties to materials that do not instinctively have these properties. In this regard, flame-retardant coatings are very important from the perspective of the exterior in order to enhance the fire resistivity of flammable materials. There are plenty of studies in this area. Pan et al. [111] studied the fabrication of montmorillonite and titanate nanotube-based coatings via a layer-by-layer self-assembly method to enhance the thermal stability, flame retardancy, and ultraviolet protection of polyethylene terephthalate (PET) fabric. The TGA results showed that the char residue was improved obviously after the sample was covered with the hybrid coating. Furthermore, four-quadlayer chitosan/MMT/chitosan/TNTs coating significantly improved the fire resistance of PET fabric, as evidenced by the obvious reduction (48% and 36%) in the peak heat-release rate and total heat release compared with those of the pure PET fabric, respectively.

Wang et al. [112] used MnO_2_ nanosheet-based multilayer coating as a flame retardant for flexible polyurethane foam. In this study MnO_2_ nanosheets were firstly successfully deposited on the surface of flexible polyurethane (FPU) foams through layer-by-layer technique for the purpose of improving their fire safety. The coating growth was realized by the alternative deposition of MnO_2_ nanosheet suspensions and polyelectrolytes solutions. Thermogravimetric analysis results showed that the thermal stability of the coated FPU foams increased obviously after the fabrication of MnO_2_ nanosheets. Moreover, the peak heat-release rate (pHRR) values of the coated FPU foams decreased remarkably with a relative low loading compared with the pure sample, indicating that the MnO_2_-based coating had tremendous advantages in reducing the fire hazards of the FPU foams.

Wang et al. [113] used a sandwich-like coating consisting of alternating montmorillonite and β-FeOOH for reducing the fire hazard of flexible polyurethane foam. In this study, the nanocoating was deposited on the surface of flexible polyurethane (FPU) foams through a layer-by-layer assembly technique. The coating growth was performed by alternately immersing FPU foams into polyethylenimine (PEI) solution, MMT-alginate suspension, and β-FeOOH dispersion. The coated FPU foams showed a lower peak heat-release rate (PHRR) compared to their counterparts fabricated via the introduction of MMT nanosheets or β-FeOOH nanorods alone. Furthermore, the concentration of volatile products released from the coated FPU foams was reduced remarkably, indicating that the binary components had tremendous advantages in reducing the flammability of the material.

Nour et al. developed a novel flame-retardant back-coating layer for historic textile fabrics. In their study, silica nanoparticles originated from agriculture-waste rice husk were prepared through a one-pot thermal method. The silica nanoparticles were further impregnated with organic borate-producing flame-retardant composite. The flame retardancy of the back-coated linen samples improved, achieving a high class of flame-retardancy for textile fabrics, with a burning rate of zero compared to 80.3 mm/min for the blank sample. They investigated the synergistic effect of flame retardancy between nanoparticles and organic borate [114].

Wang et al. [115] synthesized phosphorylated graphene oxides (PGO) using a one-pot method and these were fabricated on the surface of cotton fabrics for the improvement of fire safety. A vertical flame test and a cone calorimeter test revealed that the flame retardancy of cotton fabrics was obviously improved by PGO-based multilayer coating. A plausible flame-retardancy mechanism was proposed: PGO with large layered structures could have effectively insulated the permeation of oxygen and volatile flammable gases, thereby decreasing the heat-release rate; on the other hand, the presence of phosphorus could have played an important role in the catalytic charring effect during combustion, which significantly promoted the formation of char residue and then further prevented the permeation of oxygen and pyrolysis products. 

## 4. Conclusions

In the 21st century, scientists’ efforts are aimed at inventing effective and environmentally safe methods of extinguishing flames. One promising method appears to be acoustic. As a practical matter, for acoustic technology to be one of the common techniques for extinguishing flames, there is a need for continued research toward a thorough understanding of the ability to extinguish fires of different classes depending on the parameters of the extinguishing wave. Since it is difficult to extinguish flames using low-power sound sources, researchers note the need to use higher power. Thus, in practical applications, it is possible to increase the distance between the flame source and the extinguisher output by using high and very high acoustic power [3,13,14,32,45,46]. At the design stage of the acoustic system, it is worth considering the multipoint placement of sound sources so that the acoustic waves are directed towards the flame source. They could be installed at several locations, while one or more outputs may have resonant horn tips. This allows the acoustic flux to be directed from multiple outputs to the flame source and, by using constructive interference, the extinguishing efficiency is improved [45]. Multiple smart components, such as cameras, mobile terminals, and sensors, can be deployed in the environment, as shown in [116], among others. It becomes possible to simultaneously transmit signals from any type of measurement sensors using a transmultiplexing technique [117]. When building such a system, additional or dedicated interference screens, acoustic panels, baffles, and other elements, such as materials that absorb the harmful part of the acoustic wave energy, can be useful. This makes it possible to compensate for the losses resulting from the omnidirectional emission of the acoustic extinguisher, which affect the reducing of the distance to which effective flame extinguishing through acoustic waves has been observed. It is stated that in addition to improving fire-extinguishing methods in firefighting, it is also important to produce fire-resistant materials, which is the focus of many institutions and research centers [118,119,120,121,122]. These materials prevent the fire from spreading, creating a phenomenon that helps extinguish the fire. In particular, the production of self-extinguishing materials will greatly support fire suppression. In this sense, the importance of producing fire-resistant materials and the multidimensional signal processing method used to extinguish the fire is emphasized in this article.

## Figures and Tables

**Table 1:** Flame-retardant reactions of huntite, hydromagnesite, ATH, and MH.

Huntite:	Mg_3_Ca(CO_3_)_4_ → 3MgO + CaO + 4CO_2_
Hydromagnesite:	Mg_4_(CO_3_)_3_(OH)_2_.3H_2_O → 4MgO + 3CO_2_ + 4H_2_O
Magnesium hydroxide:	Mg(OH)_2_ → MgO + H_2_O
Aluminum hydroxide:	2Al(OH)_3_ → Al_2_O_3_ + 3H_2_O

**Table 2:** Sample codes and descriptions.

SFPlast	Uncoated plastic substrate
SFBoya	Coated with pure dye
A%1N	%1 Antimony oxide and HH coating
A%5N	%5 Antimony oxide and HH coating
A%10N	%10 Antimony oxide and HH coating
A%15N	%15 Antimony oxide and HH coating
A%30N	%30 Antimony oxide and HH coating
A%50N	%50 Antimony oxide and HH coating
B%1N	%1 Boric acid and HH coating
B%5N	%5 Boric acid and HH coating
B%10N	%10 Boric acid and HH coating
B%15N	%15 Boric acid and HH coating
B%30N	%30 Boric acid and HH coating
B%50N	%50 Boric acid and HH coating
HH: huntite/hydromagnesite mineral	

## Data Availability

Not applicable.
